# The growth factor/cytokine midkine may participate in cytokine storm and contribute to the pathogenesis of severe acute respiratory syndrome coronavirus 2-infected patients

**DOI:** 10.1186/s43168-021-00087-6

**Published:** 2021-09-25

**Authors:** Sema Ketenci, A. Şükrü Aynacıoğlu

**Affiliations:** Department of Medical Pharmacology, Faculty of Medicine, Istanbul Atlas University, Anadolu Cad. No:40, Kağıthane, 34408 Istanbul, Turkey

**Keywords:** Midkine, COVID-19, SARS-CoV-2, Cytokine storm, Inflammation

## Abstract

**Background:**

The current coronavirus disease 2019 (COVID-19) outbreak caused by severe acute respiratory syndrome coronavirus 2 (SARS-CoV-2) has emerged in Wuhan, China, and has rapidly become a global challenge, creating major challenges to health systems in almost every country in the world it has turned into a pandemic. COVID-19 poses a risky clinical situation that can range from mild illness to severe respiratory failure, requiring admission to intensive care.

**Main body:**

It is known that SARS-CoV-2 infection causes a cytokine storm in some critically ill patients. However, more and more evidence showed that there is a dramatic increase in cytokine levels in patients diagnosed with COVID-19. Midkine (MK) is involved in various physiological and pathological processes, which some of them are desired and beneficial such as controlling tissue repair and antimicrobial effects, but some others are harmful such as promoting inflammation, carcinogenesis, and chemoresistance. Also, MK is expressed in inflammatory cells and released by endothelial cells under hypoxic conditions.

**Conclusions:**

Considering all this information, there are strong data that midkine, an important cytokine known to increase in inflammatory diseases, may be overexpressed in patients who are positive for COVID-19. The overexpression of MK reveals a picture leading to fibrosis and damage in the lung. Therefore, questions arise about how the expression  of  MK  changes in COVID-19 patients and can we use it as an inflammation biomarker or in the treatment protocol in the future.

## Background

The severe acute respiratory syndrome coronavirus 2 (SARS-CoV-2), which is responsible for coronavirus disease 2019 (COVID-19), not only created an extremely important worldwide problem in health management, but also generated disturbances in societies’ economy and social and cultural structure [1]. Although, the majority of COVID-19-infected patients are asymptomatic or have “mild” symptoms, such as fever, fatigue, and dry cough, some patients can progress to severe disease manifestations, including pneumonia, pulmonary edema, vascular hyper-permeability, and acute respiratory distress syndrome (ARDS) [2]. In addition, very serious conditions such as respiratory failure, septic shock, multiple organ failure, and even death occur in approximately 5% of patients [3]. In such seriously ill COVID-19 patients, the production of pro-inflammatory mediators and cytokines, including tumor necrosis factor alpha (TNF-α), interleukin (IL) 1 beta (IL-1β), IL-6, IL-18, and interferon gamma (IFNϒ), is abnormally increased that result in a “cytokine storm,” causing a diffuse alveolar damage [4, 5]. Some symptoms attributed to these cytokines are fever, chills, headaches, dizziness, and fatigue. Additionally, these cytokines can contribute to severe pathologies such as cardiomyopathy, lung injury, and septic shock.

Midkine (MK), a low molecular-weight growth factor (a heparin-binding cytokine), is strongly expressed during embryogenesis, whereas it is downregulated to relatively low levels in healthy adults [6]. However, MK is overexpressed back in various pathologies, including inflammatory diseases and many malignancies [7, 8]. It is involved in several physiological and/or pathological cell functions, including survival, reproduction, repair, and growth, and has chemotactic activity as well as proinflammatory actions [9, 10]. Since the overproduction of pro-inflammatory mediators and cytokines plays important role in the pathogenesis of COVID-19, we propose that besides other cytokines, MK may be also overexpressed in SARS-CoV-2 infection and participate in or modulate the course of COVID-19. MK has chemotactic actions, resulting in the accumulation of inflammatory cells, such as macrophages and neutrophils, which in turn aggravate the inflammatory response [11]. Additionally, in the context of COVID-19, which uses angiotensin-converting enzyme 2 (ACE-2) receptor for its clinical manifestations, MK activates angiotensin-converting enzyme (ACE), leading to higher concentrations of Ang II, leading to impaired functions of the alveoli [12]. These and other pathophysiological functions of MK and the possible relationship to SARS-CoV-2 infection are discussed in this review.

## Main text

### Inflammation and cytokine storm in SARS-CoV-2-infected patients

Actually, cytokines attend to the normal immune response to infectious agents in healthy individuals. However, several pathogenic infections, including SARS-CoV-2, are often associated with an excessive cytokine release called cytokine storm, which result in tissue damage [13]. At the time by the binding of SARS-CoV-2 to ACE-2, which serves as a functional receptor for SARS-CoV-2, the immune system of patients becomes activated [14, 15]. This, in turn, induces the accumulation of inflammatory cells with subsequent production of pro-inflammatory cytokines and chemokines at the infection area, which can also spread to many extra-pulmonary organs [2].

### Association and interaction between RAS and COVID-19

In physiological conditions, after the biotransformation of angiotensinogen to angiotensin I (Ang I) by renin, the ACE converts Ang I to angiotensin II (Ang II), which may contribute to inflammation, fibrosis, tissue damage, and edema in the lungs. On the other hand, Ang II is converted by ACE-2 to angiotensin (1–7), which has anti-inflammatory and vasodilatory properties that balanced the effects of Ang II. However, in COVID-19, the SARS-CoV-2 interacts with the RAS through ACE-2, which is necessary for the entry of the virus into pneumocytes as well as its replication [14]. Consequently, the expression of ACE-2 is downregulated by SARS-CoV-2, causing overactivation of renin-angiotensin-system (RAS) that results in increased pulmonary vasoconstriction, edema, hypoxia, and lung damage in COVID-19 patients [16, 17].

### The effect of hypoxia and activation of hypoxia-inducible factor 1α (HIF-1α) in COVID-19

The lung, an organ exposed to a high amount of oxygen, is subject to various infections, including SARS-CoV-2. Due to fluid accumulation in the alveoli, as a result of SARS-CoV-2 invasion, the effectiveness of air exchange decreases dramatically, which then results in hypoxemia and subsequently ARDS [18, 19]. This hypoxic effect of viral occupation contributes to several pathophysiological changes in the lung and is also involved in all stages of COVID-19. Hypoxia, a powerful inflammatory stimulant, is also induced in inflammatory conditions [20–24]. Besides leading to the high amount of pro-inflammatory cytokines and the creation of cytokine storm on the infection region, hypoxia triggered simultaneously several pathophysiological processes, including induction of hypoxia-inducible factor-1α (HIF-1α). HIF-1α is expressed in certain cell types, including immune cells, and regulates cell metabolism and inflammation [25, 26]. Under normal pressure of oxygen in the bloodstream, the expression of HIF-1α caused by phagocytic cells, such as neutrophils and macrophages, is low. However, in infection sites, they increase HIF-1α expression, which in turn stimulates the expression of several pro-inflammatory cytokines [27, 28]. Owing to its proinflammatory properties, it has been suggested that inhibition of HIF-1α activity can reduce the SARS-CoV-2-related inflammation and relieve the severity of COVID-19 [29].

### NETosis, oxidative stress, ROS, and COVID-19

Several pathophysiological mechanisms occur simultaneously when SARS-CoV-2 binds to ACE-2 in the lung. Primarily, monocytes recruited into the alveolar space secrete pro-inflammatory cytokines which are responsible for cytokine storm. Additionally, recruited macrophages release also cytokines and chemokines that augmented capillary permeability, pulmonary edema, and followed by neutrophil recruitment. Increased neutrophil invasion leads to the release of neutrophil extracellular traps (NETs) that are intracellular contents such as DNA, histones, and proteins. Several studies showed that this process, called NETosis, is associated closely with COVID-19 [30–32]. The excessive neutrophil degranulation precipitates lung injury and damages the alveolar-capillary barrier. In addition, NETosis is associated with increased levels of intracellular reactive oxygen species (ROS) of neutrophils [33]. But on the other hand, ROS can destroy pathogens directly by causing oxidative damage as well as indirectly, by inducing pathogen elimination via NETs formation in neutrophils [34].

### Possible relationship between COVID-19 pathogenesis and MK

MK is involved in various physiological and pathological processes, which some of them are desired and beneficial such as controlling tissue repair and antimicrobial effects, but some others are harmful such as promoting inflammation, carcinogenesis, and chemo-resistance [35–37]. Although animal models of myocardial infarction have shown a protective role of MK for the injured cardiac tissue by its anti-apoptotic effect and its role in angiogenesis [38, 39], most studies showed that MK is harmful under chronic inflammatory conditions [40]. MK is a cytokine with strong pro-inflammatory characteristics, causing macrophage and neutrophil recruitment to the inflamed region and interact with other growth factors and cytokines, particularly TNFα. Furthermore, MK mediates and exhibits enhancement of fibrinolytic activity, which are important processes in the initial stage of inflammatory responses of various pathologies [11, 41, 42]. The expression of MK is induced by TNFα, an important component of cytokine storm in COVID-19, and vice versa [43, 44]. Therefore, it is highly possible that MK is contributed to the cytokine invasion and interacts with other cytokines and chemokines in SARS-CoV-2 infections. This pro-inflammatory effect and subsequently occurring several pathophysiological processes, in that MK is involved, could be detrimental rather than protective. In an animal study, it has been found that the expression of MK was induced in the lung endothelium of micro-vessels and alveolar-capillary endothelial cells by oxidative stress and upregulated by ACE, which hydrolyses Ang I to form Ang II. Ang II induces NADPH oxidase (Nox) expression, and the increased expression of Nox, which initiated ROS production, induced further oxidative stress, and subsequently accelerated MK and ACE generation [44]. Hypoxia, resulting from cytokine storm, inflammation, and other molecular mechanisms, including induction of hypoxia-inducible factor 1-α (HIF1-α), is an important component of SARS-CoV-2 infection [28, 45]. It has been shown that MK expression increased during hypoxia by the binding of HIF1-α to hypoxia-responsive elements located in the MK promoter [12, 46]. These findings suggest that a close relationship exists between MK, hypoxia, and HIF1-α, by enhancing each other’s pathophysiological effects. An important issue of COVID-19 is the development of ARDS, a result that may cause severe pulmonary injury and even death. Several studies demonstrated that MK is closely involved in the pathogenesis of ARDS. To clarify the possible role of the MK signaling pathway in ARDS, Zhang et al. showed that exposure to a mechanical stretch of lung epithelial cells led to an epithelial-mesenchymal transition profile associated with increased expression of ACE which was attenuated by silencing MK. Furthermore, they found out that the plasma levels of MK were higher in patients with ARDS than in healthy subjects [47]. Similarly, in idiopathic pulmonary fibrosis patients, the serum MK level was also higher compared to healthy subjects, supporting the role of MK in the development of ARDS [48]. Moreover, in midkine-deficient mice, low expression of collagen and α-smooth muscle actin, as well as a low value for the pathological lung fibrosis score, was detected. Thus, MK participates to the progression of pulmonary fibrosis, mainly by regulating inflammatory cell migration into the lung and augmenting TNF-α and tumor growth factor β (TGF-β) expression [48].

**Fig. 1:**
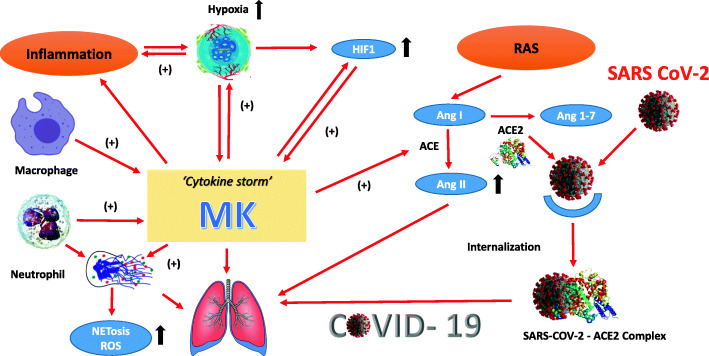
Schematic representation of hypothetic actions of midkine in relation to SARS-CoV-2 infection. SARS CoV-2, severe acute respiratory syndrome coronavirus-2; RAS, renin-angiotensin system; Ang, angiotensin; ROS, reactive oxygen species; NETs, neutrophil extracellular traps; HIF-1, hypoxia-inducible factor-1

Table 1 and Fig. 1 summarizes the possible pathophysiological mechanisms of MK in relation to COVID-19.

**Table 1:** The possible involvement of MK overexpression to SARS-CoV-2 manifestations

Manifestations of SARS-CoV-2	Pathophysiological cell functions of midkine	Reference
Pulmonary inflammation	• Recruitment of inflammatory cells • Cytokine release from neutrophils and macrophages • Enhancement of fibrinolytic activity	Takada 1997 [11] Wang 2008 [41] Zhang 2015 [47] Misa 2017 [48]
Pulmonary hypoxia	• Inducing hypoxia via inflammation • Binding of HIF1-α to hypoxia-responsive elements located in the MK promoter	Eltzschig 2011 [21] Minamino 2001 [23] Cramer 2003 [25] Walmsley 2005 [26]
Pulmonary fibrosis, ARDS	• Regulating inflammatory cell migration into the lung and induction of TNF-α and TGF-β expression • MK–RAS pathway	Zhang 2015 [47] Misa 2017 [48] You 2008 [44] Hobo 2009 [12]
Pulmonary RAS dysregulation	• Increasing pathophysiological actions of Ang II via expression of ACE	Hobo 2009 [12]

## Conclusions

Current clinical observations indicate that SARS-CoV-2 infection can range from an unapparent non-symptomatic infection to severe pulmonary damage and multiorgan failure. The excessive secretion of several cytokines is closely related to the development of clinical symptoms in COVID-19 patients. This abnormal and uncontrolled production of cytokines has been observed in most of the patients with SARS-CoV-2-related pneumonia, increasing the progression of COVID-19 and mortality. Furthermore, the overproduction of inflammatory cytokines contributes to acute lung injury and ARDS. Therefore, to improve the outcome and reduce mortality of SARS-CoV-2, cytokine monitoring is recommended for the diagnosis and treatment of COVID-19. MK, which is a cytokine and growth factor and is significantly upregulated upon exposure to various harmful stimuli, including inflammation, is likely to accompany the cytokine attack that occurs in SARS-CoV-2 infections. Drugs targeting MK, such as antibodies and RNA aptamers that suppressed the generation and/or action of MK, may be a useful part of therapeutic modalities applied in the management of COVID-19.

## Data Availability

Not applicable to this manuscript.

## Abbreviations

COVID-19: Coronavirus disease 2019
SARS-CoV-2: Severe acute respiratory syndrome coronavirus 2
MK: Midkine
ARDS: Acute respiratory distress syndrome
TNF-α: Tumor necrosis factor alpha
IL: Interleukin
ACE-2: Angiotensin-converting enzyme 2
ACE: Angiotensin-converting enzyme
Ang I: Angiotensin I
Ang II: Angiotensin II
HIF-1α: Hypoxia-inducible factor 1α
NETs: Neutrophil extracellular traps
ROS: Reactive oxygen species
Nox: NADPH oxidase
TGF-β: Tumor growth factor β
